# Haplin power analysis: a software module for power and sample size calculations in genetic association analyses of family triads and unrelated controls

**DOI:** 10.1186/s12859-019-2727-3

**Published:** 2019-04-02

**Authors:** Miriam Gjerdevik, Astanand Jugessur, Øystein A. Haaland, Julia Romanowska, Rolv T. Lie, Heather J. Cordell, Håkon K. Gjessing

**Affiliations:** 1 Department of Global Public Health and Primary Care, University of Bergen, Bergen, Norway; 2 Department of Genetics and Bioinformatics, Norwegian Institute of Public Health, Oslo, Norway; 3 Centre for Fertility and Health, Norwegian Institute of Public Health, Oslo, Norway; 4 Computational Biology Unit, University of Bergen, Bergen, Norway; 5 Institute of Genetic Medicine, Newcastle University, International Centre for Life, Central Parkway, Newcastle upon Tyne, UK

**Keywords:** Log-linear and multinomial models, Genome-wide association studies (GWAS), Statistical power estimation, Sample size estimation, Haplin, EMIM

## Abstract

**Background:**

Log-linear and multinomial modeling offer a flexible framework for genetic association analyses of offspring (child), parent-of-origin and maternal effects, based on genotype data from a variety of child-parent configurations. Although the calculation of statistical power or sample size is an important first step in the planning of any scientific study, there is currently a lack of software for genetic power calculations in family-based study designs. Here, we address this shortcoming through new implementations of power calculations in the **R** package Haplin, which is a flexible and robust software for genetic epidemiological analyses. Power calculations in Haplin can be performed analytically using the asymptotic variance-covariance structure of the parameter estimator, or else by a straightforward simulation approach. Haplin performs power calculations for child, parent-of-origin and maternal effects, as well as for gene-environment interactions. The power can be calculated for both single SNPs and haplotypes, either autosomal or X-linked. Moreover, Haplin enables power calculations for different child-parent configurations, including (but not limited to) case-parent triads, case-mother dyads, and case-parent triads in combination with unrelated control-parent triads.

**Results:**

We compared the asymptotic power approximations to the power of analysis attained with Haplin. For external validation, the results were further compared to the power of analysis attained by the EMIM software using data simulations from Haplin. Consistency observed between Haplin and EMIM across various genetic scenarios confirms the computational accuracy of the inference methods used in both programs. The results also demonstrate that power calculations in Haplin are applicable to genetic association studies using either log-linear or multinomial modeling approaches.

**Conclusions:**

Haplin provides a robust and reliable framework for power calculations in genetic association analyses for a wide range of genetic effects and etiologic scenarios, based on genotype data from a variety of child-parent configurations.

**Electronic supplementary material:**

The online version of this article (10.1186/s12859-019-2727-3) contains supplementary material, which is available to authorized users.

## Background

Statistical power or sample size analysis is an essential first step in the planning of any scientific study. Such analyses ensure that a study is capable of answering its stated research questions and are a prerequisite for optimal study design [1]. Furthermore, a power analysis is required in most research proposals. Statistical power calculations are particularly important in genome-wide association studies (GWAS) in order to maximize the scientific gains from the typically high genotyping and assay costs. Moreover, GWAS are often underpowered due to the large number of single-nucleotide polymorphisms (SNPs) being assessed, leading to issues of multiple testing. Most effect sizes reported from genetic association studies of complex traits are small [2–4], which further limits the power. The statistical power of a study affects the interpretation of the results. Low power may result in a high number of false negatives, and a power analysis might elucidate whether negative findings were the result of the study being underpowered.

Log-linear and multinomial modeling are closely related approaches that offer a flexible framework for genetic association analysis. Both approaches enable the estimation of genetic effects in addition to hypothesis testing. Beyond the standard case-control design, they are capable of incorporating child, parent-of-origin (PoO) and maternal effects based on genotype data from case-parent triads, as well as a range of other child-parent configurations. They can also handle incomplete triad data as well as independent controls. Moreover, the models are readily extended to haplotype analysis. Due to these appealing features, there has been much interest in the application of log-linear or multinomial models in genetic association studies [5–12], and the models are implemented in well-established software packages such as Haplin [10, 13] and EMIM (Estimation of Maternal, Imprinting and interaction effects using Multinomial modelling) [11, 12].

General-purpose software tools for statistical power and sample size analysis are not set up to handle the genetic study designs and effect estimates available from case-parent triads with unrelated controls. Although there are tools that offer power calculations for some genetic association studies, e.g., Quanto [14–16] and Genetic Power Calculator (GPC) [17], a comprehensive framework for power analysis based on the full triad design is lacking.

We propose a complete setup for power calculations tailored to binary disease traits, which we have implemented as a new module in the **R** package Haplin [10, 13]. In the new implementations, a power analysis can be performed based on the asymptotic variance-covariance structure of the parameter estimator or by a simulation procedure. The power for child, PoO, maternal, and gene-environment (GxE) effects are easily estimated. Haplin also enables power analyses for haplotypes, taking into account unknown SNP phase. The calculations can be performed for both autososomal and X-linked markers, and a variety of study designs can be accommodated.

Our paper is structured as follows. In the “Implementation” section, we first introduce the Haplin software and briefly present our new power calculation approaches. We then provide a short tutorial on power calculations for child, PoO and maternal effects, focusing on the use of asymptotic approximations. In the “Results” section, we illustrate our power calculations for a wide range of scenarios. We also compare our asymptotic power approximations to the powers attained by Haplin and EMIM in simulations, thus confirming the equivalent inference provided by log-linear and multinomial modeling. In Additional file 1, we derive the variance-covariance matrix underlying the asymptotic power calculations. Furthermore, because the Haplin framework includes numerous features for power analysis, we provide a more detailed and extensive tutorial, including power analysis for GxE interactions, in Additional file 2. In addition, we outline some of the possibilities for power calculations under different X-chromosome models, and we also show how the power calculations can be extended to haplotype analysis. Finally, we show the flexibility of our simulation approach, demonstrating different parameterization models and study designs.

## Implementation

Our power calculation tool has been added to the **R** package Haplin, which provides an extensive framework for genetic epidemiological analyses of binary traits. The new power calculation module has been integrated into the original setup for genetic association analysis in Haplin and is based on log-linear modeling, as previously described by Gjessing and Lie [10]. Haplin implements a full maximum-likelihood model for estimation and computes explicit estimates of relative risks with asymptotic standard errors and confidence intervals. It enables the estimation of child, PoO and maternal effects, as well as interactions between these genetic effects and categorical or ordinal exposure variables (i.e., GxE) [18, 19]. Haplin also incorporates analyses of X-linked markers in a straightforward manner, and different X-chromosome models may be fitted depending on the desired underlying assumptions [20–22]. In Haplin, the main unit of study is the case-parent triad, in which affected children and both of their biological parents are genotyped. However, the log-linear model can be extended to include independent control children or control triads in a hybrid design, under the “rare disease” assumption [23]. Note that unrelated controls are optional but not required, because “pseudo-controls” can be constructed from the non-transmitted parental alleles in case-parent triads [24–27]. The expectation maximization (EM) algorithm [28] is implemented in Haplin to account for unknown parental origin in ambiguous (uninformative) triads. Additionally, the EM algorithm accounts for missing information on certain individuals, such as when some triads are reduced to child-mother dyads due to missing data on the father. Although the fundamental model in Haplin relates to a single multi-allelic marker, it extends directly to haplotypes over multiple markers by statistically reconstructing haplotypes of unknown phase [10]. Furthermore, because the calculations can be performed in parallel, genome-wide association analyses are readily accommodated. The log-linear model in Haplin assumes Hardy-Weinberg equilibrium (HWE), Mendelian transmission and random mating. A detailed description of the underlying model is provided in several of our previous publications [10, 18, 29].

### Genetic effects and study designs

**Fig. 1:**
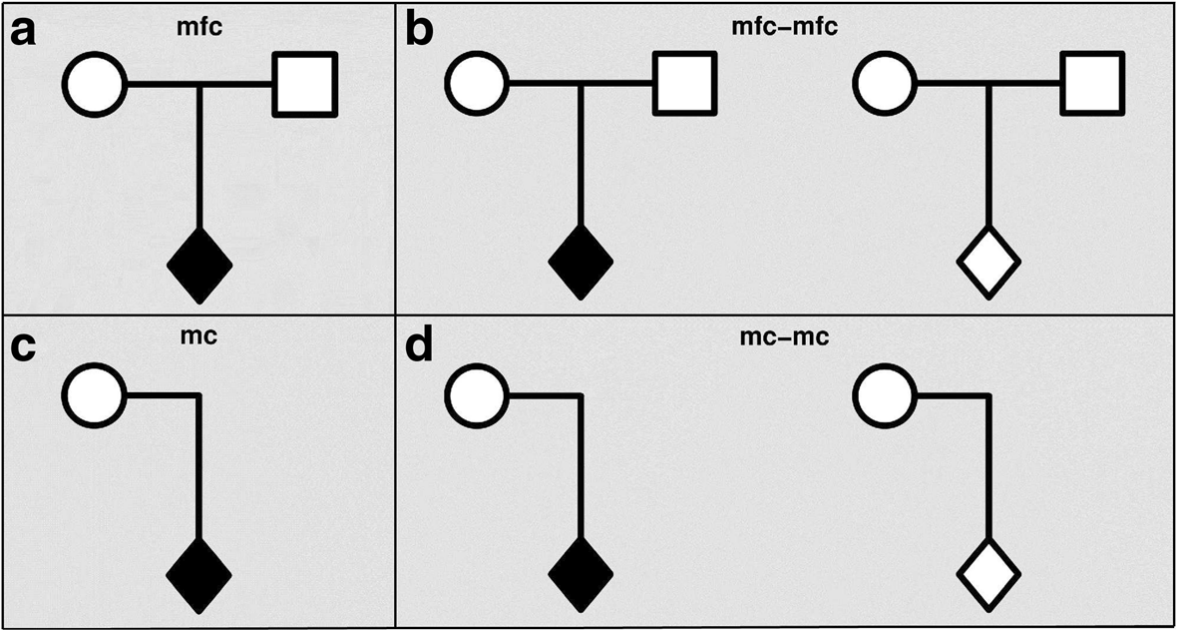
A selection of designs for genetic association studies: **a** Case-parent triad (mfc); **b** Case-parent triad with independent control-parent triad (mfc-mfc); **c** Case-mother dyad (mc); **d** Case-mother dyad with independent control-mother dyad (mc-mc)

Within the Haplin framework, based on the log-linear modeling approach, we have developed a new and complete module for performing power calculations. The basic calculations relate to child, PoO and maternal effects, and our definitions of these genetic effects are provided in Table 1. The power depends on the underlying penetrance models, i.e., the probability of a child exhibiting the disease conditional on a particular genetic composition, which we define in Table 2. A variety of child-parent configurations are available for power analysis in Haplin, and a small selection of the possible study designs is shown in Fig. 1. We use the following abbreviations to describe the family designs. We let the letters c, m and f denote a child, mother and a father, respectively. Thus, mfc denotes a case-parent triad, and mc denotes a case-mother dyad. Moreover, mfc-mfc denotes the full hybrid design, whereas mc-mc denotes the hybrid design consisting of case-mother and unrelated control-mother dyads. The possible configurations in Haplin also include designs such as c-c (the standard case-control design), fc (case-father dyad), mfc-mc (case-parent triad with unrelated control-mother dyad) and mfc-mf (case-parent triad with unrelated control parents). The full list of supported study designs are provided on the Haplin website [13].

**Table 1:** Genetic effects

Effects	Description
Child	A variant allele may increase the risk of a disease only when carried by an individual himself/herself. We refer to this as a “child effect” since it is frequently estimated from the offspring in a case-parent triad. However, the individual referred to as a child might be of any age, depending on the phenotype of interest, and the same effect can also be estimated in case-control studies.
Parent-of-origin (PoO)	A PoO effect occurs if the effect of a variant allele in the child depends on whether it is inherited from the mother or the father. In statistical terms, we define a PoO effect as the interaction effect RRR=RR_*M*,*j*_/RR_*F*,*j*_, which is a measure of the risk increase (or decrease) associated with allele *A*_*j*_, when derived from the mother as opposed to the father. In contrast, regular child-effect analyses assume that the effect of an allele in the child is independent of parental origin. Note that genomic imprinting (an epigenetic phenomenon where one of the inherited parental alleles is expressed whereas the other is silenced) may cause PoO effects [32].
Maternal	A mother’s genotype may influence fetal development directly, for example through maternal metabolic factors operating in utero [33], and may affect health throughout life [34]. A maternal effect occurs when a variant allele carried by the mother increases the risk of disease in her child, regardless of whether or not the allele has been transferred to the child [35]. This is distinct from child and PoO effects, in which we measure the effect of alleles in the child himself/herself. Because these underlying genetic mechanisms lead to entirely different biological interpretations, distinguishing between the genetic effects is particularly important in advancing the understanding of the etiology underlying a complex disease [11, 36, 37].

**Table 2:** Parameterization of penetrances

Effects	Parameterization of penetrances	
Child	$ B\cdot \text {RR}_{j}\text {RR}_{l}\text {RR}_{jl}^{*} $	(1)
Parent-of-origin (PoO)	$ B\cdot \text {RR}_{M,j}\text {RR}_{F,l}\text {RR}_{jl}^{*} $	(2)
Child and maternal	$ B\cdot \text {RR}_{j}\text {RR}_{l}\text {RR}_{jl}^{*} \cdot \text {RR}_{i}^{(M)}\text {RR}_{j}^{(M)}\text {RR}_{ij}^{(M)*} $	(3)
PoO and maternal	$ B\cdot \text {RR}_{M,j}\text {RR}_{F,l}\text {RR}_{jl}^{*} \cdot \text {RR}_{i}^{(M)}\text {RR}_{j}^{(M)}\text {RR}_{ij}^{(M)*} $	(4)

*B* is the baseline risk level, typically associated with the (more common) reference allele; RR_*j*_ is the risk increase associated with allele *A*_*j*_, relative to *B*; RR_*M*,*j*_ and RR_*F*,*j*_ are the relative risks associated with allele *A*_*j*_, depending on whether the allele is transmitted from the mother or the father; the double-dose parameter $\text {RR}_{jl}^{*}$ measures the deviation from what would be expected in a multiplicative dose-response relationship, i.e., $\text {RR}_{jl}^{*}=\text {RR}_{j}^{*}$ when *j*=*l* and $\text {RR}_{jl}^{*}=1$ when *j*≠*l*; $\text {RR}_{i}^{(M)}$ is the relative risk associated with allele *A*_*i*_ carried by the mother, and $\text {RR}_{ij}^{(M)*}$ is the maternal double-dose parameter, interpreted analogously to $\text {RR}_{ij}^{*}$. To ensure that the model is not overparameterized, we set RR=1 for the reference allele

### Power calculations in Haplin

In this section, we demonstrate how to perform basic power calculations in Haplin, implemented in the function hapPowerAsymp. The power is computed analytically through asymptotic approximations, scaled to the appropriate sample size. We apply the asymptotic normal distribution of the log-scale parameter and use the chi-squared non-centrality parameter of the Wald test. The variance-covariance matrix is computed from a log-linear model which accounts for transmission ambiguities and missing data; its derivation is provided in Additional file 1. The theory underlying our asymptotic power calculations is outlined in more detail elsewhere [29].

In Haplin, the asymptotic power calculations are easy to perform. In general, one only needs to specify the study design and its sample size, the allele frequencies, and the type of genetic effect and its magnitude. Table 3 shows example Haplin commands for estimating the power for child, PoO and maternal effects. In all examples, we calculate the power for a diallelic SNP, using 500 case-parent triads. The study design is specified by the arguments cases and controls, using the notation from Fig. 1. Thus, 500 case-parent triads are specified by the argument cases=c(mfc=500), whereas 500 case-mother dyads would be specified by cases=c(mc=500). A hybrid design consisting of 200 case-mothers dyads and 500 control-parent triads would be expressed by the combination cases=c(mc=200) and controls=c(mfc=500).

**Table 3:** Examples of asymptotic power calculations in Haplin

Effects	Haplin commands	Output
a) Child	hapPowerAsymp(cases = c(mfc=500),	$haplo.power
	haplo.freq = c(0.8,0.2),	Haplotype RR.power
	RR = c(1,1.4))	1 ref
		2 0.88
b) PoO	hapPowerAsymp(cases = c(mfc=500),	$haplo.power
	haplo.freq = c(0.8,0.2),	Haplotype RRcm.power RRcf.power RRcm_cf.power
	RRcm = c(1,2), RRcf = c(1,1.5))	1 ref ref ref
		2 1 0.87 0.48
c) Child and maternal	hapPowerAsymp(cases = c(mfc=500),	$haplo.power
	haplo.freq = c(0.8,0.2),	Haplotype RR.power RRm.power
	RR = c(1,1.4), RR.mat = c(1,1.2))	1 ref ref
		2 0.9 0.42
d) PoO and maternal	hapPowerAsymp(cases = c(mfc=500),	$haplo.power
	haplo.freq = c(0.8,0.2),	Haplotype RRcm.power RRcf.power RRcm_cf.power RRm.power
	RRcm = c(1,2), RRcf = c(1,1.5),	1 ref ref ref ref
	RR.mat = c(1,1.2))	2 0.99 0.65 0.2 0.17

The power is calculated for a diallelic SNP, using 500 case-parent triads (cases = c(mfc=500)), and a MAF of 0.2 (haplo.freq = c(0.8,0.2)). The argument RR specifies the relative risk associated with the child effect, whereas the power to detect a PoO effect is calculated by replacing RR by the two relative risk arguments RRcm and RRcf, which refer to the parental origin of the allele carried by the child. Maternal effects can be included by adding the maternal relative risk parameter RR.mat to the original child or PoO command. Note that the order of alleles to which the relative risk parameters refer corresponds to the order used for the haplotype frequencies. Here, the less frequent allele is set as the risk allele and the more frequent allele is used as reference. The nominal significance level defaults to 0.05, but other values can be specified by the argument alpha

The genetic effects are determined by the choice of relative risk parameter(s), which also specifies the effect sizes. Corresponding to the parameterization model in Eq. (1) (defined in Table 2), a child effect is specified by the relative risk argument RR (Table 3a). Allele frequencies are specified by the argument haplo.freq. Note that the order and length of the specified relative risk parameter vectors should always match the corresponding allele frequencies. All examples assume a minor allele frequency (MAF) of 0.2. Thus, from Table 3a we see that the power is 88% when the less frequent allele at a diallelic marker is associated with a relative risk of 1.4, as expressed by the combination of allele frequencies haplo.freq=c(0.8,0.2) and relative risks RR=c(1,1.4). By default, the more frequent allele is chosen as reference (Table 3a, first row of the Haplin output).

As illustrated in Table 3b, the power to detect a PoO effect is computed by replacing the argument RR by the two relative risk arguments RRcm and RRcf, denoting parental origin m (mother) and f (father). Both RR_*M*_ and RR_*F*_ are estimated freely, and individual tests for the null hypotheses RR_*M*_=1 and RR_*F*_=1 are constructed. The corresponding power estimates are denoted by RRcm.power and RRcf.power, respectively. In addition, we are interested in testing the actual PoO effect, estimated by comparing the maternally and paternally derived effects by the ratio RRR=RR_*M*_/RR_*F*_. The null hypothesis of RRR=RR_*M*_/RR_*F*_=1 means no PoO effect, and the power to detect the PoO effect is output as RRcm_cf.power, here estimated to be 48% when RRcm = c(1,2) and RRcf = c(1,1.5). For more details on PoO testing and its relationship to imprinting, see Gjerdevik et al. [29].

Since children and their mothers have an allele in common, a maternal effect might be statistically confounded with a child or a PoO effect. Corresponding to the parameterization models in Eq. (3) and (4) (Table 2), the power of a maternal effect can be analyzed jointly with that of a child effect or a PoO effect by adding the relative risk argument RR.mat to the original child or PoO model (Table 3c and d). The resulting power estimates control for the possible confounding of these effects with one another. When adjusting for the maternal effect in Table 3c, the power to detect the child effect is 90%. Conversely, when adjusting for the child effect, the power to detect the maternal effect is 42%. The example in Table 3d, involving joint PoO and maternal effects, has a similar interpretation.

In Table 3, the nominal significance level defaults to 5%. However, other values can be specified by using the argument alpha. The current implementation of hapPowerAsymp does not allow deviations from the multiplicative dose-response assumption. Thus, the double-dose parameters RR^∗^ and RR^(*M*)∗^ (Eq. 1-4 in Table 2) are equal to 1 and do not need to be specified in the Haplin command. However, we expect future versions of hapPowerAsymp to handle power calculations for separate single- and double-dose effects.

### Power simulations in Haplin

Haplin also includes an extensive setup for power calculation through simulations. Simulation approaches are robust ways of checking software implementations, attained power, and attained significance level. They are particularly useful for small to moderately sized datasets, in which the asymptotic properties of the log-linear model might not hold true. In these situations, the extent and direction of the possible bias can best be assessed using simulations. In Haplin, power simulations are carried out using a two-step approach, by applying the functions hapRun and hapPower. First, hapRun simulates haplotype data, in which triad genotypes are generated from the multinomial distribution. The multinomial probabilities are computed by listing all possible genotype combinations in the triad format and then applying the sampling model described in Gjessing and Lie [10]. hapRun then performs Haplin runs, i.e., statistical inference, on the simulated data. To speed up these calculations, hapRun allows for parallel processing. In the second step, the simulation results from hapRun are submitted to hapPower, which computes the power by calculating the fraction of p-values less than the nominal significance level.

Clearly, the asymptotic power approximation is much more time-efficient than brute-force simulations; in its current implementation, however, it is somewhat more restricted. The simulation approach is completely general; it enables power calculations for a wider range of parameterization models, such as deviations from the multiplicative dose-response assumption. The simulation approach also handles a wider array of child-parent configurations and allows for missing individuals to be generated at random. Examples and relevant Haplin commands are provided in Additional file 2.

## Results

### Examples of asymptotic power calculations

**Fig. 2:**
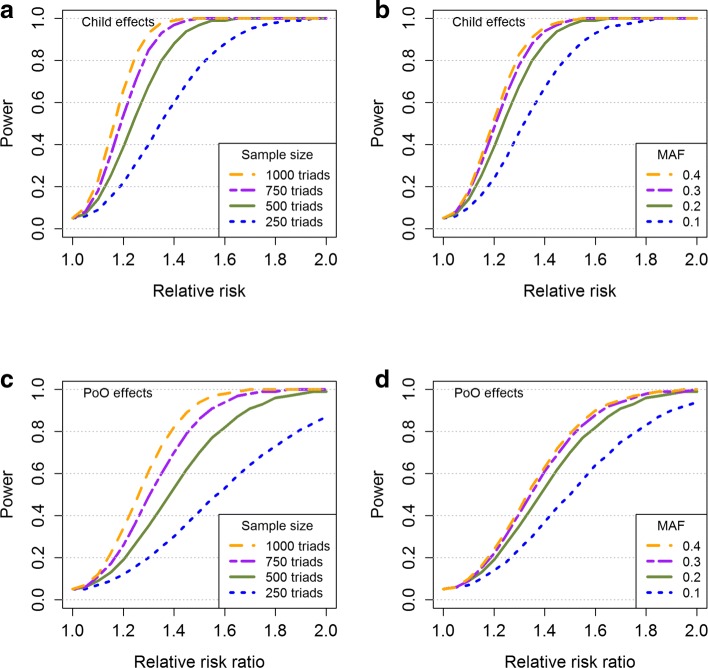
Power analysis using the Haplin function hapPowerAsymp. **a** Child effects for varying numbers of case-parent triads, using a MAF of 0.2; **b** Child effects for varying values of MAFs, using a total of 500 case-parent triads; **c** PoO effects for varying numbers of case-parent triads, using a MAF of 0.2; **d** PoO effects for varying values of MAFs, using a total of 500 case-parent triads. For the PoO effects, RR_*F*_=1, so that the value of RR_*M*_/RR_*F*_ is equal to RR_*M*_. A nominal significance level of 0.05 was used throughout. The power was calculated at relative risks/relative risk ratios of 1,1.05,1.10,…,2. Intermediate values correspond to line segments joining two adjacent points

We illustrate the use of our power function hapPowerAsymp by plotting power curves for different scenarios, as shown in Fig. 2. Power calculations for child effects are shown in panels **a** and **b**, and power calculations for PoO effects are shown in panels **c** and **d**. For the PoO effects, we set RR_*F*_=1, so that the value of RRR=RR_*M*_/RR_*F*_ is equal to the value of RR_*M*_. In the left panels (**a** and **c**), we used varying numbers of case-parent triads and a MAF of 0.2. In the right panels (**b** and **d**), the power was calculated using varying MAFs and a total of 500 case-parent triads. We used a nominal significance level of 5% throughout.

In all panels, the green, solid line represents scenarios in which 500 case-parent triads and a MAF of 0.2 were used. For child effects, we have 80% power to detect an RR of 1.35. However, using 250 case-parent triads, the corresponding power decreases to 51% (panel **a**). Moreover, with 500 case-parent triads and a MAF of 0.1, the power to detect an RR of 1.35 is 57% (panel **b**). The PoO analysis can be viewed as a statistical interaction. Compared with the child-effect analysis, a higher sample size is therefore required for the PoO analysis to reach the same statistical power for a similar effect size. Approximately 1200 case-parent triads are needed to detect an RRR of 1.35 with 80% power and a MAF of 0.2 (panel **c**). With 500 case-parent triads and a MAF of 0.2, we have approximately 80% power to detect an RRR of 1.6. Using a MAF of 0.1, the corresponding power is 64% (panel **d**).

Note that sample size and power are directly related measures. For given relative risks, power curves similar to Fig. 2 can be made with sample size on the x-axis.

### Comparison of the asymptotic power approximations to the simulated power in Haplin and EMIM

Similar to Haplin, the command line software PREMIM and EMIM are easy-to-use tools for the estimation of child, PoO and maternal effects based on genotype data from a number of different study designs [11, 12]. PREMIM generates required input files for EMIM by extracting the required genotype data from standard-format pedigree data (PLINK) files [30], and EMIM performs the subsequent statistical analyses. PREMIM and EMIM are written in C++ and FORTRAN 77, respectively, and are therefore considerably faster than **R** implementations. EMIM allows a variety of different parameterization models, which makes it an appealing software for power comparisons with Haplin. Because EMIM uses multinomial modeling, its inference should be similar to that of Haplin [31]. However, to account for unknown parental origin in ambiguous (uninformative) triads or dyads, EMIM maximizes the multinomial likelihood directly (via a direct search algorithm), whereas Haplin maximizes the likelihood using the EM algorithm.

We compared the asymptotic power calculations in Haplin to the power attained by Haplin and EMIM in data simulations. The asymptotic power was computed using the function hapPowerAsymp, whereas the simulated power in Haplin was calculated using hapRun and hapPower. EMIM performs genetic association analyses, but corresponding power calculations are not implemented. To calculate the power attained by EMIM, we first used the Haplin function hapSim to simulate the genotype data. The data was then converted to the standard PLINK-format files, which were subsequently fed into PREMIM and EMIM. Given that the power calculations in Haplin are based on the Wald test, we also used the Wald test for inference in EMIM. Lastly, we calculated the fraction of p-values less than the nominal significance level. We analyzed child, PoO and maternal effects employing the parameterizations presented in Table 2, assuming a multiplicative dose-response model. We simulated data for a variety of child-parent configurations (mfc, mc, mfc-mfc, mc-mc), with effect sizes ranging between 1.0 and 2.0, and a MAF of 0.2. We based the power comparisons on 500 case families in each design, i.e., 500 case-mother dyads or 500 case-parent triads, reflecting that the number of case children available is often a constraint when designing a study. For the hybrid designs, we added an equal number of unrelated control families. The simulations were based on 10,000 replicates of data for a single SNP, and we used a nominal significance level of 0.05. HWE and random mating were assumed throughout.

**Fig. 3:**
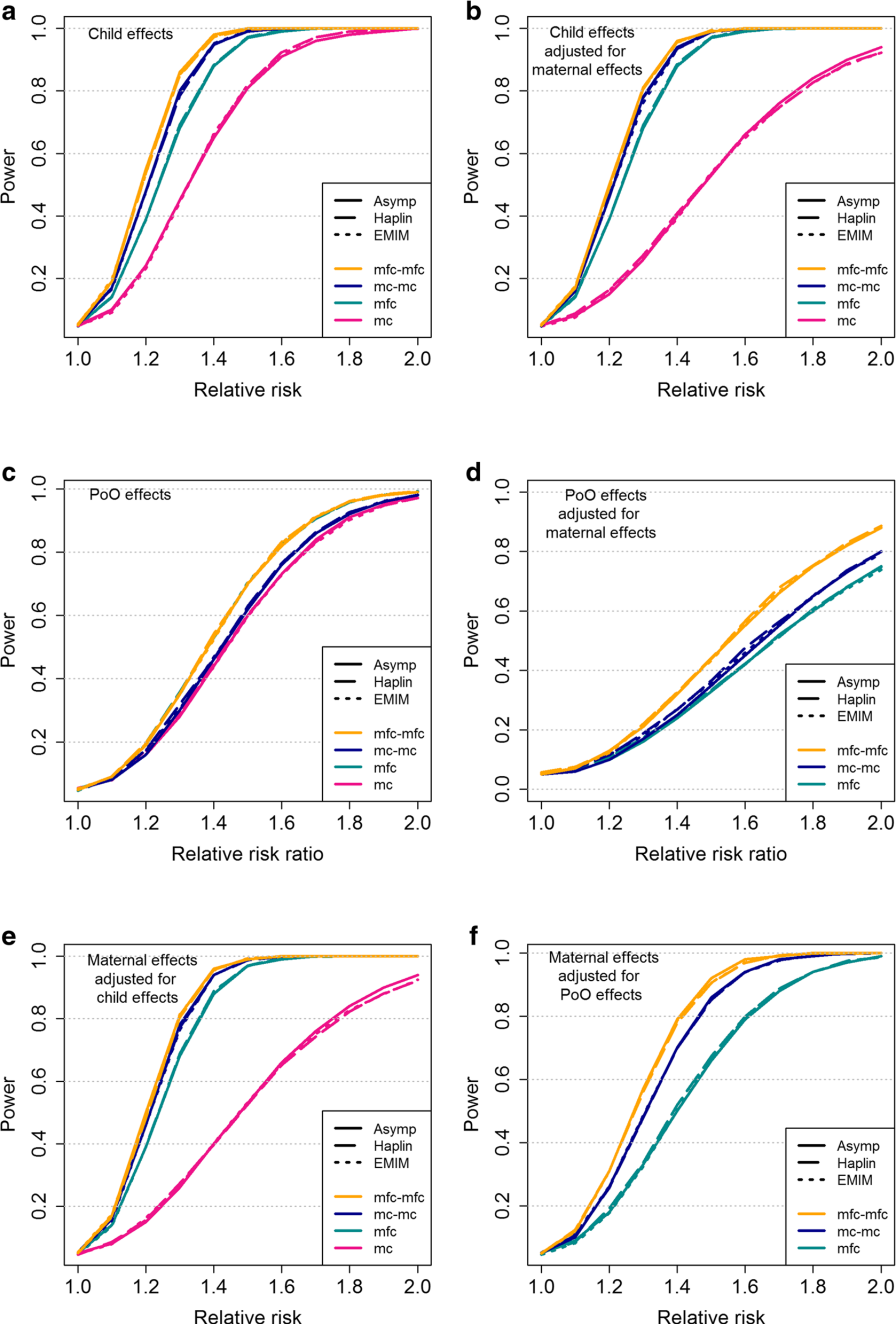
Comparison of the asymptotic power calculations with the power attained by Haplin and EMIM in data simulations. The power was calculated for different child-parent configurations, assuming a MAF of 0.2 and a nominal significance level of 0.05. The results were based on 500 case families and, when applicable, 500 unrelated control families. All simulations were based on 10,000 replicates of data for a single SNP. **Asymp**: Power calculations in Haplin, based on asymptotic approximations (Haplin function hapPowerAsymp); **Haplin**: Power calculations in Haplin, based on data simulations. The power is the proportion of tests rejected by Haplin (Haplin functions hapRun and hapPower); **EMIM**: Power calculations based on data simulations in Haplin (Haplin function hapSim). The power is the proportion of tests rejected by EMIM. **a** Child effects (RR >1); **b** Child effects, adjusting for maternal effects (RR >1 and RR^(*M*)^=1); **c** PoO effects (RR_*M*_/RR_*F*_>1 and RR_*F*_=1); **d** PoO effects, adjusting for maternal effects (RR_*M*_/RR_*F*_>1 and RR_*F*_=RR^(*M*)^=1); **e** Maternal effects, adjusting for child effects (RR^(*M*)^>1 and RR = 1); **f** Maternal effects, adjusting for PoO effects (RR^(*M*)^>1 and RR_*M*_=RR_*F*_=1). The power was calculated at relative risks/relative risk ratios of 1,1.1,1.2,…,2. Intermediate values correspond to line segments joining two adjacent points. Note that for all study designs, the power was calculated based on asymptotic approximations in Haplin, as well as simulations where both Haplin and EMIM were used to analyze the genetic data. The lines for Asymp, Haplin and EMIM are nearly overlapping, demonstrating consistent results

The results are shown in Fig. 3. Child effects are displayed in panels **a** and **b**, and PoO effects are displayed in panels **c** and **d**, with panels **b** and **d** showing the results obtained when the child and PoO effects were calculated while adjusting for possible maternal effects (even though, in the simulation model, we did not assume maternal effects, i.e., we set RR^(*M*)^=1). For the PoO effects, we set RR_*F*_=1, so that the value of RR_*M*_/RR_*F*_ is equal to the value of RR_*M*_. Panels **e** and **f** show the power to detect maternal effects, while adjusting for possible child or PoO effects (simulated under models where no such child or PoO effects existed, i.e., RR^(*M*)^>1 and RR=1, and RR^(*M*)^>1 and RR_*M*_=RR_*F*_=1, respectively).

Note that panels **b** and **e** are equivalent because the power to detect a given child or maternal effect is identical when adjusting for possible confounding of the effects with one another. However, this symmetry depends on the study design and will not necessarily hold if case-mothers are unavailable for genotyping (results not shown). PoO effects are essentially estimated in case families, by contrasting the frequencies of alleles transmitted from mother to child with those of alleles transmitted from father to child. Thus, unrelated control families do not add extra power to the case-parent triad design, as can be seen from the overlapping results of the mfc and mfc-mfc designs in panel **c**. Note that we excluded the mc design from the joint PoO and maternal effect-analyses (panels **d** and **f**) because the penetrance model in Eq. (4) (Table 2) would become overparameterized. Overall, Fig. 3 shows that the results are highly consistent between the asymptotic power approximations and the simulated power in Haplin and EMIM, demonstrating that the asymptotic power function performs well when the asymptotic properties underlying the log-linear model hold true. Furthermore, the consistency between Haplin and EMIM across a wide spectrum of genetic scenarios confirms the computational accuracy of the inference methods used in both programs. Altogether, the results indicate that Haplin provides a robust and reliable framework for power calculations in genetic association studies when the genetic analyses are based on either log-linear or multinomial modeling.

## Conclusions

To our knowledge, a comprehensive software for power analysis based on the full triad design has been lacking. Here, we have developed and showcased extensive, new and easy-to-use functionalities for statistical power analyses based on log-linear modeling, incorporated in the **R** package Haplin. In Haplin, power analysis can be carried out analytically using the asymptotic variance-covariance structure of the parameter estimator, or, by a straightforward simulation procedure. The two approaches for power calculations complement each other, balancing time efficiency against generality. Haplin enables power calculations to be performed for child, PoO, maternal and GxE effects, based on genotype data from a variety of family-based study designs. An inherent strength of the Haplin framework is its ability to compute power for both single SNPs and haplotypes, either autosomal or X-linked. We plan to continue to expand the present framework for power analysis, adding new features for power calculations as additional methods for genetic association analysis are developed and incorporated into the Haplin software.

To facilitate power analysis in Haplin, we have provided relevant example commands in Table 3. In addition, an extended tutorial is provided in Additional file 2, demonstrating power analysis for GxE interactions, X-linked models and haplotype effects, as well as our simulation functions hapRun and hapPower. Researchers can easily apply our functions using arguments and parameter values relevant to their own data.

The standard Haplin implementation assumes haplotype-frequency parameters under HWE instead of a model with all mating-type parameters [5, 6]. This improves power and facilitates haplotype reconstruction. The triad design itself protects against population stratification, but some of that benefit is lost if HWE is not fulfilled. However, top hits from a GWAS analysis can be checked retrospectively for HWE. As for power calculations, a full set of mating-type frequencies will seldom be available prior to study start, and a HWE assumption simplifies the calculations.

We conducted a thorough comparison of the asymptotic approximation approach with the power attained by Haplin and EMIM in data simulations. Child, PoO and maternal effects were assessed. The concordant results obtained confirm the computational accuracy of the inference methods used in both programs. They also demonstrate that power calculations in Haplin are applicable to genetic association studies analyzed by either log-linear or multinomial modeling approaches. Thus, Haplin provides a robust and reliable framework for power calculations in genetic association analyses for various genetic effects and etiologic scenarios, based on genotype data from a wide range of different child-parent configurations.

## Availability and requirements

**Project name:** Haplin

**Project home page:** https://people.uib.no/gjessing/gene-tics/software/haplin

**Operating system(s)**: Platform independent

**Programming language:** Haplin is implemented as a standard package in the statistical software **R**. It is available from the official **R** package archive, CRAN (https://cran.r-project.org).

**Other requirements:** None

**License:** GPL (>= 2)

**Any restrictions to use by non-academics:** None

Information on EMIM and PREMIM is available from https://www.staff.ncl.ac.uk/richard.howey/emim.

## Additional files

Additional file 1 An asymptotic approximation of **Σ**. (PDF 184 kb)

Additional file 2 Power and sample size calculations in Haplin. (PDF 369 kb)

## Abbreviations

EM algorithm: The expectation maximization algorithm
GxE: Gene-environment interaction
GWAS: Genome-wide association study
HWE: Hardy-weinberg equilibrium
MAF: Minor allele frequency
mc: The case-mother dyad design
mc-mc: Case-mother dyads with unrelated control-mother dyads
mfc: The case-parent triad design
mfc-mfc: Case-parent triads with unrelated control-parent triads
PoO effect: Parent-of-origin effect
RR: Relative risk
RRR: Relative risk ratio
SNP: Single-nucleotide polymorphism
