# Supplementary material for: Haplin power analysis: a software module for power and sample size calculations in genetic association analyses of family triads and unrelated controls
Source: BMC Bioinformatics. 2019 Apr 2;20:165. doi: 10.1186/s12859-019-2727-3 (PMC6444579; doi:10.1186/s12859-019-2727-3)
Supplement: Supplementary file 1 — An asymptotic approximation of Σ. (PDF 184 kb) [file 12859_2019_2727_MOESM1_ESM.pdf]

Additional file 1 — An asymptotic approximation of $\boldsymbol{\Sigma}$

Likelihood model Assume a locus has l different alleles. Typically, $l = 2$ at a single SNP. With k SNPs, there are $l = 2^k$ different possible haplotypes, each considered an allele at the locus, assuming no recombination between SNPs. For a mother-father-child triad, the genotype of the triad can be written as $(A_{M1}A_{M2}, A_{F1}A_{F2}, A_{C1}A_{C2})$, where A_M denotes the maternal alleles, A_F denotes the paternal alleles, and A_C denotes the alleles of the child. Assume that the parental alleles are ordered in such a way that the second allele is transmitted to the child; i.e., we have $A_{C1} = A_{M2}$ and $A_{C2} = A_{F2}$. This permits a more compact notation with the full triad as an ordered quadruplet $(A_{M1}, A_{M2}, A_{F1}, A_{F2})$.

To list all possible triad genotypes, we construct a 4-column matrix $\boldsymbol{G}$ with one column for each of the parental alleles, including all possible allele combinations. For instance, for a diallelic SNP with alleles 1 and 2,

$$\boldsymbol{G} = \begin{array}{c} \begin{matrix} A_{M1} & A_{M2} & A_{F1} & A_{F2} \end{matrix} \\ \begin{pmatrix} 1 & 1 & 1 & 1 \\ 2 & 1 & 1 & 1 \\ 1 & 2 & 1 & 1 \\ 2 & 2 & 1 & 1 \\ 1 & 1 & 2 & 1 \\ 2 & 1 & 2 & 1 \\ \vdots & \vdots & \vdots & \vdots \\ 2 & 2 & 2 & 2 \end{pmatrix} \end{array}.$$

The matrix $\boldsymbol{G}$ has dimensions $q \times 4$, where $q = l^4$. In particular, $q = 2^{4k}$ when considering a locus with k diallelic SNPs, where the alleles are the 2^k possible haplotypes at the locus.

Assuming the full genotype of all triads could be observed, the log-linear model assumes that the number of triads $\boldsymbol{n} = [n_1, \dots, n_q]$, corresponding to the rows of $\boldsymbol{G}$, can be described by independent Poisson distributions, where

$$\boldsymbol{m} = \exp(\boldsymbol{X\beta})$$

is a $q \times 1$ vector of the expected number of triads in each row, $\boldsymbol{\beta}$ is a $p \times 1$ parameter vector, $\boldsymbol{X}$ is a $q \times p$ design matrix (described in more detail below), and the exponential function is computed elementwise. We assume that $\boldsymbol{1} \in \text{colspace}(\boldsymbol{X})$, where $\boldsymbol{1} = [1, \dots, 1]^T$. If $\hat{\boldsymbol{\beta}}$ is the maximum likelihood estimator derived from this model, and $\hat{\boldsymbol{m}} = \exp(\boldsymbol{X}\hat{\boldsymbol{\beta}})$, the condition

$$\hat{\boldsymbol{m}}^T \boldsymbol{1} = \boldsymbol{n}^T \boldsymbol{1}$$

holds [1, Chapter 10], i.e., the sum of the expected number of triads is equal to the sample size $N = \boldsymbol{n}^T\boldsymbol{1}$. Let $m_{\boldsymbol{\cdot}} = \boldsymbol{m}^T\boldsymbol{1}$, and define $\boldsymbol{p} = \boldsymbol{m}/m_{\boldsymbol{\cdot}}$, i.e., the cell probabilities.

Each row in $\boldsymbol{G}$ corresponds to a fully observed triad genotype, that is, a triad can be associated with a specific row of $\boldsymbol{G}$ only if the alleles of the mother, father, and child are all fully known. From observed data, however, one will often obtain triads where, for instance, the genotypes of the father is lacking. Also, since the alleles at the locus will typically consist of haplotypes derived from a sequence of SNPs, the unknown phase of the SNPs will represent an ambiguity regarding the triad alleles. For any observed triad j, we define $\boldsymbol{a}_j$ to be the $q \times 1$ "ambiguity vector" for triad j. To determine $\boldsymbol{a}_j$ for a given triad, we first identify all rows of $\boldsymbol{G}$ that are compatible with the observed genotype of the triad. For instance, at a SNP, if we observe a mother with genotype (1 2), a child with the genotype (2 2), and the father is missing, the full triad could be either (1 2, 1 2, 2 2) or (1 2, 2 2, 2 2), which correspond to rows 11 and 15, respectively, in the $\boldsymbol{G}$ matrix. The ambiguity vector $\boldsymbol{a}_j$ is then a vector with ones at positions 11 and 15, and zeros otherwise. Similarly, with two or more SNPs, unknown haplotype phase introduces ambiguities which are incorporated in the ambiguity vector $\boldsymbol{a}_j$.

Let $\mathcal{A}$ be the set of all possible ambiguity vectors, i.e., those corresponding to all observed genotypes. Note that $\boldsymbol{a_j}$ is a many-to-one mapping from the rows of $\boldsymbol{G}$ into $\mathcal{A}$, and thus $P(\boldsymbol{a_j} = \boldsymbol{a}) = \boldsymbol{a}^T\boldsymbol{p}$, i.e., the sum over all row probabilities compatible with the observed genotype.

Design matrix Using $\boldsymbol{G}$ as the starting point, the corresponding $q \times p$ design matrix $\boldsymbol{X}$ for a log-linear model can be derived, including columns for estimating allele frequencies, child allele dose effects, etc. The form of $\boldsymbol{X}$ will depend on what model is being estimated in a given instance. For example, to estimate the child relative risk RR_2 associated with allele 2, we first create two dummy vectors 1_M and 1_F. The dummy 1_M is set to one when the A_{M2} column of $\boldsymbol{G}$ is equal to 2, and zero otherwise. Similarly, 1_F is set to one when the A_{F2} column is 2, and zero otherwise. That is, 1_M and 1_F indicate whether the child inherited allele 2 from the mother and/or the father, respectively. The design matrix $\boldsymbol{X}$ should then contain a column equal to $1_M + 1_F$, and $\hat{\mathrm{RR}}_2 = \exp(\hat{\beta})$, where $\hat{\beta}$ is the estimated parameter corresponding to this column. This choice would entail $\mathrm{RR}_1 = 1$ and $\mathrm{RR}^*_{2,2} = 1$, i.e., a multiplicative response model with allele 1 as the reference allele. If the model should allow deviations from the multiplicative response, including $1_M \cdot 1_F$ in the $\boldsymbol{X}$ matrix would provide an estimate of $\mathrm{RR}^*_{2,2}$. By similar constructions, all models described in this paper are covered. The exact form of the $\boldsymbol{X}$ matrix is not important for the likelihood derivation below.

The asymptotic variance-covariance matrix In our likelihood model, we write $l_N(\boldsymbol{\beta}) = \log(L_N(\boldsymbol{\beta}))$ for the log-likelihood based on N triads. Let $\hat{\boldsymbol{\beta}}_N$ be the corresponding maximum likelihood estimator of the $p \times 1$ parameter vector $\boldsymbol{\beta}$. As described above, the $\boldsymbol{\beta}$ parameter vector contains information about haplotype frequencies and relative risks; typically, $\beta_i = \log(\mathrm{RR}_i)$ for some component i of the vector, where RR_i is the relative risk associated with

haplotype h_i. We denote the asymptotic $p \times p$ variance-covariance matrix by $\boldsymbol{\Sigma}$. From standard likelihood theory,

$$\sqrt{N}(\hat{\boldsymbol{\beta}}_N - \boldsymbol{\beta}) \xrightarrow{d} \mathcal{N}(\mathbf{0}, \boldsymbol{\Sigma})$$

as $N \to \infty$ [1, Chapter 10]. The matrix $\boldsymbol{\Sigma}$ is given as the inverse of the expected information matrix, $\mathcal{I}(\boldsymbol{\beta})$, with element (i,j) defined as

$$-E\left\{\frac{\partial^2 l(\boldsymbol{\beta})}{\partial\beta_i\partial\beta_j}\right\},$$

where $l(\boldsymbol{\beta})$ is the log-likelihood function [2].

If N is the total number of observed triads, then N is Poisson distributed with expected value equal to the sum over all rows, i.e., $m_{\bullet}$. Conditional on N, the number of triads corresponding to a row in $\boldsymbol{G}$ (if they were fully observed) follows a multinomial distribution with cell probabilities $\boldsymbol{p}$. Hence, the likelihood contribution from a single observed (possibly ambiguous) triad j is $\boldsymbol{a}_j^T\boldsymbol{p}$, and the full likelihood, accounting for ambiguities, is

$$L(\boldsymbol{\beta}) \propto m_{\bullet}{}^N e^{-m_{\bullet}} \prod_{j=1}^{N} \boldsymbol{a}_j^T \boldsymbol{p}.$$

The corresponding log-likelihood function is then

$$l(\boldsymbol{\beta}) = \sum_j (\log(\boldsymbol{a}_j^T\boldsymbol{m})) - m_{\bullet}.$$

Applying the rules for vector differentials [3], we have that

$$\partial l(\boldsymbol{\beta}) = (\sum_j \boldsymbol{b}_j^T - \boldsymbol{m}^T)\boldsymbol{X}\partial\boldsymbol{\beta},$$

where

$$\boldsymbol{b}_j = \frac{\mathrm{diag}(\boldsymbol{a}_j)\boldsymbol{m}}{\boldsymbol{a}_j^T\boldsymbol{m}}.$$

Furthermore, the second derivative of the log-likelihood function is

$$\partial^2 l(\boldsymbol{\beta}) = (\partial\boldsymbol{\beta})^T\boldsymbol{X}^T\left(\mathrm{diag}(\sum_j \boldsymbol{b}_j) - \sum_j \boldsymbol{b}_j\boldsymbol{b}_j^T - \mathrm{diag}(\boldsymbol{m})\right)\boldsymbol{X}(\partial\boldsymbol{\beta}).$$

Consequently, the observed Fisher information matrix is

$$I_N(\boldsymbol{\beta}) = \boldsymbol{X}^T\left(\mathrm{diag}(\boldsymbol{m}) - \mathrm{diag}(\sum_j \boldsymbol{b}_j) + \sum_j \boldsymbol{b}_j\boldsymbol{b}_j^T\right)\boldsymbol{X},$$

and as $N \to \infty$,

$$\frac{1}{N}I_N(\boldsymbol{\beta}) \sim \boldsymbol{X}^T\left(\mathrm{diag}(\boldsymbol{p}) - \mathrm{diag}(E(\boldsymbol{b})) + E(\boldsymbol{b}\boldsymbol{b}^{\boldsymbol{T}})\right)\boldsymbol{X}.$$

It follows that the asymptotic variance-covariance matrix of $\hat{\boldsymbol{\beta}}_N$ is

$$\boldsymbol{\Sigma} = \left[\boldsymbol{X}^T \left(\mathrm{diag}(\boldsymbol{p}) - \mathrm{diag}(E(\boldsymbol{b})) + E(\boldsymbol{b}\boldsymbol{b}^{\boldsymbol{T}})\right) \boldsymbol{X}\right]^{-1},$$

and thus

$$\mathrm{var}(\hat{\boldsymbol{\beta}}_N) \sim \frac{1}{N}\boldsymbol{\Sigma}.$$

References

[1] Christensen R. Log-linear models and logistic regression. 2nd ed. New York, NY: Springer; 1997.

[2] Pawitan Y. In all likelihood. Oxford: Clarendon Press; 2001.

[3] Wand MP. Vector differential calculus in statistics. The American Statistician. 2002;56(1):55–62.
