# Supplementary material for: Haplin power analysis: a software module for power and sample size calculations in genetic association analyses of family triads and unrelated controls
Source: BMC Bioinformatics. 2019 Apr 2;20:165. doi: 10.1186/s12859-019-2727-3 (PMC6444579; doi:10.1186/s12859-019-2727-3)
Supplement: Supplementary file 2 — Power and sample size calculations in Haplin. (PDF 369 kb) [file 12859_2019_2727_MOESM2_ESM.pdf]

Additional file 2 — Power and sample size calculations in Haplin

Haplin includes a complete setup for power calculations, extending beyond the single-SNP analyses of child, PoO and maternal effects. Here, we provide an extensive tutorial and illustrate power analyses for a range of relevant genetic and etiologic scenarios. Nevertheless, this tutorial is not intended as an exhaustive documentation of the power framework and its functions. We therefore strongly recommend consulting the **R** help page, which includes detailed and up-to-date information on the power functions and all their arguments. As we continue to expand our framework for power analysis, changes to the presented commands may occur. Such updates will be documented on the Haplin website at `https://people.uib.no/gjessing/genetics/software/haplin`, as well as on the **R** help page.

The supplementary material is structured as follows. We start with an introduction of the asymptotics-based functions `snpPower` and `snpSampleSize`, before continuing with an extended tutorial of `hapPowerAsymp`. The two last sections are devoted to `hapRun` and `hapPower`, which calculate the power by using simulations.

`snpPower` and `snpSampleSize`

For single-SNP analyses of child effects, statistical power and sample size calculations are most easily done with the Haplin functions `snpPower` and `snpSampleSize`. `snpPower` computes the power for a single SNP by counting the number of "real" case alleles (transmitted alleles from case triads), "real" control alleles (all alleles from control triads) and pseudo-control alleles (non-transmitted alleles from case families). A multiplicative dose-response relationship is assumed. `snpPower` calculates the power by using the asymptotic normal approximation for the natural logarithm of the odds ratio (the relative risks and odds ratios are used interchangeably due to the "rare disease assumption"). It computes the power for a given number of case families, control families, relative risks, minor allele frequencies (MAFs) and type I error rates. For example, to compute the power for 200 case-parent triads and 100 control children, assuming a relative risk of 1.4, a minor allele frequency of 0.2, and a nominal significance level of 5%, use the command

```
snpPower(cases=list(mfc=200), controls=list(c=100), RR=1.4, MAF=0.2, alpha=0.05).
```

In `snpPower`, the power can be calculated for a mixture of different family designs and for several combinations of the input variables simultaneously. Please refer to the **R** help page and the Haplin website for an explanation of the arguments and its options.

Note that most of the functionality of `snpPower` is covered by the more flexible Haplin function `hapPowerAsymp`, which also extends to power analyses of haplotype effects, parent-of-origin (PoO) effects, maternal effects, gene-environment interactions (GxE), etc. However, `snpPower` is somewhat easier to apply and is therefore useful for simple power calculations of single-SNP child effects.

`snpSampleSize` is the inverse function of `snpPower`. For child effects, it computes the number of case and control families required for a single SNP to attain the desired power for specified family designs and given values of relative risks, minor allele frequencies and type I error rates. Examples and documentation are given on the **R** help page and on the Haplin website.

`hapPowerAsymp`: Extensions to X-linked markers, gene-environment interactions and haplotype effects

Basic power calculations of child, PoO and maternal effects using `hapPowerAsymp` are described in the main article. Here we show how to extend power analyses to X-linked markers, GxE, and haplotype effects.

X-linked markers

Genetic association analyses of X-linked markers might be of particular relevance if the prevalence of a complex trait or disease is systematically different for males versus females. Various X-chromosome models are implemented in Haplin. The models depend on the underlying assumptions regarding allele-effects in males versus females, which may include sex-specific baseline risks, shared or distinct relative risks for males and females, as well as X-inactivation in females. A detailed description of the parameterization models is provided in our previous studies [1, 2, 3]. Corresponding power analyses are readily available in `hapPowerAsymp`, and an example of X-chromosome power analysis is shown in Table S1a. In addition to the arguments needed to perform power calculations of child, maternal or PoO effects on autosomal markers, three arguments are required to specify an X-linked penetrance model. The argument `xchrom` must be set to `TRUE`, which indicates power analysis of X-chromosome markers. Furthermore, the argument `sim.comb.sex` specifies how to deal with sex differences on the X-chromosome. We have used the option `single`, which means that the effect of one (single) allele in males equals the effect of a single allele dose in females. However, the default value is `double`, which corresponds to X-inactivation; a single allele in males has the same effect as one of the two alleles in homozygous females, assuming that the other allele is inactivated. The argument `BR.girls` gives the ratio of baseline risk for females relative to males. In the example of Table S1a, we assumed a ratio of 1, i.e., the same baseline risk in females and males.

Gene-environment interactions

A gene-environment interaction occurs when a genetic effect is modified by an environmental exposure. For example, maternal alcohol consumption, cigarette smoking or vitamin intake in the periconceptional period might modify the association between SNPs and a birth defect [4, 5]. The genetic effect in question might be a child, PoO or maternal effect. In Haplin, interactions between a genetic effect and a categorical exposure variable are incorporated into the log-linear framework by fitting the log-linear model separately for each exposure stratum. We then apply a Wald test to assess whether the relative risk estimates differ significantly across exposure levels [6, 7]. In `hapPowerAsymp`, the power to detect a GxE effect is automatically computed when the number of strata is larger than 1, specified by the argument `n.strata`. Each of the stratum-specific arguments `cases`, `controls`, `haplo.freq`, `RR`, `RRcm`, `RRcf` and `RR.mat` are given as lists. Their lengths should be equal to the number of strata, and each element of the list specifies the argument for one stratum. An example of GxE power analysis of child effects and two exposure strata is given in Table S1b. We used 500 case-parent triads in the first stratum and 300 case-parent triads in the second (`cases = list(c(mfc=500),c(mfc=300))`). The list format is, however, only needed for arguments that vary across strata. Here we assumed that the allele frequencies are the same in both strata, and the list format is therefore redundant (`haplo.freq = c(0.8,0.2)`). There are no associations in the first stratum, whereas the minor allele is associated with the disease in the second stratum (`RR = list(c(1,1),c(1,1.4))`).

Haplotypes

By default, `hapPowerAsymp` performs power calculations for a diallelic SNP. However, the extension to haplotypes is straightforward but requires a basic understanding of how the haplotypes are generated in Haplin. The number of markers and haplotypes is determined by the vector `nall`, where the number of markers is equal to `length(nall)`, and the number of different haplotypes is equal to `prod(nall)`. Thus, two diallelic markers are denoted by `nall = c(2,2)`, whereas a single marker with four alleles is denoted by `nall = 4`. The haplotypes are determined by creating all possible haplotypes from the given markers, in a sequence where the first marker varies most quickly. For instance, if `nall = c(3,2)`, there are six haplotypes in total. Taken in order, the haplotypes are 1-1, 2-1, 3-1, 1-2, 2-2, and 3-2. If `haplo.freq = c(0.3,0.05,0.1,0.1,0.2,0.25)` and `RR = c(1,2,1,1,1,1)`, haplotype 2-1 has a twofold risk compared to the rest of the haplotypes. Table S1c shows a haplotype example with two diallelic markers. Compared with the reference (by default the most frequent haplotype), all haplotypes are associated with an increased risk of disease. The power to detect the effect of an individual haplotype is calculated by analyzing that specific haplotype separately against the reference, using the Wald test with one degree of freedom. Here, the individual power estimates range between 63% and 74%. We also calculate the overall power, i.e., the power to detect any difference among the haplotypes, by analyzing the haplotypes jointly. With a total of four haplotypes, the Wald test has three degrees of freedom, and the power is approximately 84%.

The power calculations can be extended to three or more markers at a locus in a similar manner. An example of three diallelic SNPs (eight haplotypes) is provided in Table S1d.

Other effects

The power analyses in Table S1 were calculated for child effects. However, the power to detect PoO effects is readily computed by replacing the relative risk argument `RR` by `RRcm` and `RRcf`, similar to the example in Table 3b of the main article. For instance, in the GxE example (Table S1b), replacing `RR` with `RRcm=list(c(1,1),c(1,1))` and `RRcf=list(c(1,1),c(1,2))` would mean that there is no risk associated with the allele transmitted from the mother in either stratum, whereas the paternally derived allele is associated with the disease only in the second stratum. Maternal effects are included by adding the argument `RR.mat` (see Table 3c and d of the main article for examples).

Table S1 Asymptotic power calculations in Haplin

Etiologic scenarios	Haplin commands	Output	
a) X-chromosome	`hapPowerAsymp(cases = c(mfc=500),`<br>`haplo.freq = c(0.8,0.2), RR = c(1,1.2),`<br>`xchrom = T, sim.comb.sex = "single",`<br>`BR.girls = 1)`	`$haplo.power`<br>`Haplotype RR.power`<br>`1 ref`<br>`2 0.3`	
b) GxE	`hapPowerAsymp(n.strata = 2,`<br>`cases = list(c(mfc=500),c(mfc=300)),`<br>`haplo.freq = c(0.8,0.2),`<br>`RR = list(c(1,1), c(1,1.4)))`	`$haplo.power`<br>`Haplotype RR.power`<br>`1 ref`<br>`2 0.47`	
c) Haplotype effects, two diallelic markers	`hapPowerAsymp(nall=c(2,2), cases = c(mfc=500),`<br>`haplo.freq = c(0.4,0.3,0.2,0.1),`<br>`RR = c(1,1.3,1.4,1.5))`	`$haplo.power`<br>`Haplotype RR.power`<br>`1-1 ref`<br>`2-1 0.63`<br>`1-2 0.74`<br>`2-2 0.73`	`$overall.power`<br>`child`<br>`0.8370423`
d) Haplotype effects, three diallelic markers	`hapPowerAsymp(nall=c(2,2,2), cases = c(mfc=500),`<br>`haplo.freq = c(0.25,0.2,0.1,0.1,0.05,0.1,0.1,0.1),`<br>`RR = c(1,1.2,1,1,1.2,1,1.7,1))`	`$haplo.power`<br>`Haplotype RR.power`<br>`1 1-1-1 ref`<br>`2 2-1-1 0.24`<br>`3 1-2-1 0.05`<br>`4 2-2-1 0.05`<br>`5 1-1-2 0.12`<br>`6 2-1-2 0.05`<br>`7 1-2-2 0.89`<br>`8 2-2-2 0.05`	`$overall.power`<br>`child`<br>`0.7416111`

The power is calculated for a) X-chromosome models; b) GxE effects; c) Haplotype effects, two diallelic markers; d) Haplotype effects, three diallelic markers. The argument `cases` determines the study design and its sample size, and the argument `RR` specifies the relative risk associated with a child effect. Note that the order of alleles to which the relative risk parameters refer corresponds to the order used for the haplotype frequencies in argument `haplo.freq`. The most frequent allele/haplotype is used as reference. The arguments `xchrom`, `sim.comb.sex` and `BR.girls` are specific for power analyses of X-linked markers. The argument `n.strata` determines the number of strata and is required for power analyses of GxE effects. In c) and d), the number of markers and haplotypes is specified by the vector `nall`. Because the default value is 2 (corresponding to a diallelic SNP), this argument was not explicitly expressed in examples a) and b). The nominal significance level defaults to 0.05, but other values can be specified by the argument `alpha`

Introduction to hapRun and hapPower

The function **hapRun** simulates genotype data and performs the subsequent statistical inference. The results can then be fed to hapPower, which calculates the actual power. Because hapRun performs both the simulations and the subsequent statistical inference, the aimed target effects must be specified in addition to the simulation-specific parameters. The commands therefore require knowledge of the functions haplin and haplinStrat, which perform the statistical inference within hapRun.

We demonstrate the power simulation functions hapRun and hapPower by using the same scenarios as in Table 3 of the main article, in addition to the GxE example in Table S1. The examples are shown in Table S2. Additional to the arguments provided in hapPowerAsymp, hapRun requires the arguments nall, RRstar and response to be specified (RRstar.mat must also be specified in order to simulate maternal effects). The vector nall specifies the number of markers and haplotypes. In hapPowerAsymp, nall has the default value 2, whereas in hapRun the argument must be given explicitly. Moreover, hapRun handles deviations from the multiplicative dose-response relationship. Such deviations can be simulated by the arguments RRstar and RRstar.mat, which correspond to the parameters RR^* and $\mathrm{RR}^{(M)*}$ in Eq (1-4) from Table 2 in the main article. The target effect is specified by the haplin argument response, which has the option "mult" for estimating a multiplicative dose-response relationship, and the option "free" for estimating separate single-dose and double-dose effects. In Table S2, we *simulate* and *test* a multiplicative dose-response relationship throughout (RRstar = c(1,1), RRstar.mat = c(1,1) and response = "mult"). However, if one were to forget response = "mult", the simulated data (following a multiplicative dose-response relationship), when fed to haplin, would be used to estimate separate single-dose and double-dose effects, corresponding to the haplin default value "free".

In hapRun, the arguments RRcm and RRcf must be specified in order to *simulate* PoO effects. However, to *test* for PoO effects, one also needs to specify poo = TRUE in hapRun (Table S2b). It is thus possible to simulate PoO effects without actually testing them. The same is true for maternal effects (Table S2c and d); the arguments RR.mat and RRstar.mat enable simulations of maternal effects, but this effect is not tested unless maternal = TRUE in hapRun.

The argument hapfunc specifies which Haplin function to run on the simulated data in hapRun. Because most genetic association analyses are conducted using the function haplin, hapfunc = "haplin" is the default value. However, GxE effects are analyzed using haplinStrat, as shown in Table S2e. We recommend consulting the **R** help files for a thorough description of these Haplin functions and for further information on the target effects and the arguments to be passed onto haplin and haplinStrat.

Right now the output of hapPower contains more information than the output of hapPowerAsymp. The first result column, overall.power, displays the power for detecting an overall difference between the null model (no effects) and the full model. Whereas the other results of hapPower are based on the Wald test, the overall result is based on the likelihood ratio test. RRdd.power and RRmdd.power show the power to detect a double-dose child effect or a double-dose maternal effect, respectively. Because we have assumed a multiplicative dose-response relationship, the power to detect a double-dose child or maternal effect equals the power to detect a single-dose effect. The multiplicative double-dose PoO effect is interpreted analogously to the multiplicative double-dose child effect but is estimated by stratifying on parental origin.

Table S2 Simulated power in Haplin

Effects	Haplin commands	Output

a) Child

```
res.child <- hapRun(nall = 2, cases = c(mfc=500),
        haplo.freq = c(0.8,0.2),
        RR = c(1,1.4), RRstar = c(1,1),
        response = "mult")

hapPower(res.child)
```

```
The power was calculated using 1000 of 1000 files
  haplos overall.power RR.power RRdd.power
       1         0.878      ref        ref
       2         0.878    0.877      0.877
```

b) PoO

```
res.PoO <- hapRun(nall = 2, cases = c(mfc=500),
        haplo.freq = c(0.8,0.2),
        RRcm = c(1,2), RRcf = c(1,1.5),
        RRstar = c(1,1), poo = T,
        response = "mult")

hapPower(res.PoO)
```

```
The power was calculated using 1000 of 1000 files
  haplos overall.power RRcm.power RRcf.power
       1         0.999        ref        ref
       2         0.999      0.999      0.839

RRcm_RRcf.power RRdd.power
            ref        ref
          0.482      0.999
```

c) Child and maternal

```
res.childmat <- hapRun(nall = 2, cases = c(mfc=500),
        haplo.freq = c(0.8,0.2), RR = c(1,1.4),
        RRstar = c(1,1), RR.mat = c(1,1.2),
        RRstar.mat = c(1,1), maternal = T,
        response = "mult")

hapPower(res.childmat)
```

```
The power was calculated using 1000 of 1000 files
  haplos overall.power RR.power RRdd.power
       1         0.921      ref        ref
       2         0.921    0.899      0.899

RRm.power RRmdd.power
      ref         ref
    0.419       0.419
```

d) PoO and maternal

```
res.PoOmat <- hapRun(nall = 2, cases = c(mfc=500),
        haplo.freq = c(0.8,0.2), RRcm = c(1,2),
        RRcf = c(1,1.5), RRstar = c(1,1),
        RR.mat = c(1,1.2), RRstar.mat = c(1,1),
        maternal = T, poo = T, response = "mult")

hapPower(res.PoOmat)
```

```
The power was calculated using 1000 of 1000 files
  haplos overall.power RRcm.power RRcf.power
       1             1        ref        ref
       2             1      0.995      0.637

RRcm_RRcf.power RRdd.power RRm.power RRmdd.power
            ref        ref       ref         ref
          0.209      0.999     0.164       0.164
```

e) GxE

```
res.GxE <- hapRun(nall = 2, n.strata = 2,
        cases = list(c(mfc=500),c(mfc=300)),
        haplo.freq = c(0.8,0.2),
        RR = list(c(1,1), c(1,1.4)),
        RRstar = c(1,1), response = "mult",
        hapfunc = "haplinStrat")

hapPower(res.GxE)
```

```
The power was calculated using 1000 of 1000 files
child
0.484
```

We simulate the power for a diallelic SNP, using a MAF of 0.2 (`haplo.freq = c(0.8,0.2)`). The study design and the corresponding sample size are determined by the argument `cases`. The arguments `RR` and `RRstar` specify the relative risks associated with the child effect. Note that a multiplicative dose-response model is simulated by `RRstar = c(1,1)` and tested by the argument `response = "mult"`. The power to detect a PoO effect is calculated by replacing `RR` by the two relative risk arguments `RRcm` and `RRcf`, which refer to the parental origin of the allele carried by the child. In addition, we need to include the target effect by the argument `poo = T`. A maternal effect can be simulated by adding the maternal relative risk parameters `RR.mat` and `RRstar.mat` to the original child or PoO command, and the effect is tested by adding `maternal = T`. Power calculations of GxE effects are conducted by adding the arguments `n.strata` and `hapfunc = "haplinStrat"`, as well as specifying the stratum-specific arguments. Note that the order of alleles to which the relative risk parameters refer corresponds to the order used for the haplotype frequencies. Here, the less frequent allele is set as the risk allele, and the more frequent allele is used as reference. The nominal significance level defaults to 0.05, but different levels can be specified by the argument `alpha`. By default, `hapRun` simulates 1000 replicates of data files. Other values can be set by the argument `n.sim`. As `hapRun` simulates genotype data, the results will vary, and the precision depends on the number of replicates. The simulation procedure is time-consuming. However, one can speed up the calculations by using parallel processing, specified by the argument `cpus`

Extended family designs and missing individuals

The simulation procedure handles a variety of child-parent configurations. As shown in Table S3a, designs such as case-parent triads and case-mothers dyads may be combined. The argument `controls` can be extended in a similar manner. Moreover, if genotype data are missing at random, e.g., due to failed genotyping, missing case or control individuals can be generated at random through the arguments `gen.missing.cases` and `gen.missing.controls`. If the arguments are single numbers between 0 and 1, missing data are generated at random with these proportions for all case and controls individuals. In Table S3b, 10% of all case individuals (mothers, fathers and children) are missing. If the arguments are vectors of length equal to the number of markers, missing data are generated at random with the corresponding proportions for each marker. The arguments can also be matrices with the number of rows equal to the number of markers and three columns. Each row corresponds to a single marker, and the columns correspond to mothers, fathers and children, respectively. Thus, `gen.missing.cases = matrix(c(0,0.2,0),nrow=1)` simulates haplotype data in which 20% of the case fathers are missing at random. To ensure that the data are simulated correctly, it might be worthwhile to look at the simulated files. The argument `dire = "sim"` saves the simulated files to the given directory. If there is a large number of simulated files, a test run should be performed with a small number of data replicates (specified by the argument `n.sim`). We also note that the function `hapSim` can be used to simulate genotype data in Haplin format, without performing the actual testing.

Table S3 Simulated power in Haplin- extended family designs and missing individuals

	Haplin commands	Output
a) Extended family designs	`res.study.design <- hapRun(nall = 2,`<br>`cases = c(mfc=400, mc=100),`<br>`haplo.freq = c(0.8,0.2),`<br>`RR = c(1,1.4), RRstar = c(1,1),`<br>`response = "mult")`<br><br>`hapPower(res.study.design)`	`The power was calculated using 1000 of 1000 files`<br>`haplos overall.power RR.power RRdd.power`<br>`1 0.879 ref ref`<br>`2 0.879 0.878 0.878`
b) Missing individuals	`res.gen.missing <- hapRun(nall = 2,`<br>`cases = c(mfc=500),`<br>`gen.missing.cases = 0.1,`<br>`haplo.freq = c(0.8,0.2),`<br>`RR = c(1,1.4), RRstar = c(1,1),`<br>`response = "mult")`<br><br>`hapPower(res.gen.missing)`	`The power was calculated using 1000 of 1000 files`<br>`haplos overall.power RR.power RRdd.power`<br>`1 0.826 ref ref`<br>`2 0.826 0.825 0.825`

The power is simulated for a diallelic SNP, using different child-parent configurations and a MAF of 0.2 (`haplo.freq = c(0.8,0.2)`). In a), we combine 400 case-parent triads and 100 case-mother dyads to create one dataset (`cases = c(mfc=400, mc=100)`). In b), we simulate genotype data in which 10% of all case individuals are missing at random (`gen.missing.cases = 0.1`). The arguments `RR` and `RRstar` specify the relative risks associated with a child effect. A multiplicative dose-response model is simulated by `RRstar = c(1,1)` and tested by the argument `response = "mult"`. Note that the order of alleles to which the relative risk parameters refer corresponds to the order used for the haplotype frequencies. Here, the less frequent allele is set as the risk allele, and the more frequent allele is used as reference. The nominal significance level defaults to 0.05, but different levels can be specified by the argument `alpha`. By default, `hapRun` simulates 1000 replicates of data files. Other values can be set by the argument `n.sim`. As hapRun simulates genotype data, the results will vary, and the precision depends on the number of replicates. The simulation procedure is time-consuming. However, one can speed up the calculations by using parallel processing, specified by the argument `cpus`

References

[1] Jugessur A, Skare Ø, Lie RT, Wilcox AJ, Christensen K, Christiansen L, et al. X-linked genes and risk of orofacial clefts: evidence from two population-based studies in Scandinavia. PLoS One. 2012;7(6):e39240.

[2] Skare Ø, Gjessing HK, Gjerdevik M, Haaland ØA, Romanowska J, Lie RT, et al. A new approach to chromosome-wide analysis of X-linked markers identifies new associations in Asian and European case-parent triads of orofacial clefts. PLoS One. 2017;12(9):e0183772.

[3] Skare Ø, Lie RT, Haaland ØA, Gjerdevik M, Romanowska J, Gjessing HK, et al. Analysis of parent-of-origin effects on the X chromosome in Asian and European orofacial cleft triads identifies associations with *DMD*, *FGF13*, *EGFL6*, and additional loci at Xp22.2. Front Genet. 2018;9:25.

[4] Haaland ØA, Jugessur A, Gjerdevik M, Romanowska J, Shi M, Beaty TH, et al. Genome-wide analysis of parent-of-origin interaction effects with environmental exposure (PoOxE): an application to European and Asian cleft palate trios. PLoS One. 2017;12(9):e0184358.

[5] Haaland ØA, Lie RT, Romanowska J, Gjerdevik M, Gjessing HK, Jugessur A. A genome-wide search for gene-environment effects in isolated cleft lip with or without cleft palate triads points to an interaction between maternal periconceptional vitamin use and variants in *ESRRG*. Front Genet. 2018;9:60.

[6] Skare Ø, Jugessur A, Lie RT, Wilcox AJ, Murray JC, Lunde A, et al. Application of a novel hybrid study design to explore gene-environment interactions in orofacial clefts. Ann Hum Genet. 2012;76(3):221–236.

[7] Gjerdevik M, Haaland ØA, Romanowska J, Lie RT, Jugessur A, Gjessing HK. Parent-of-origin-environment interactions in case-parent triads with or without independent controls. Ann Hum Genet. 2018;82(2):60–73.
